# Zoledronic acid in metastatic osteosarcoma: encouraging progression free survival in four consecutive patients

**DOI:** 10.1186/s13569-016-0046-2

**Published:** 2016-04-28

**Authors:** Robert M. Conry, Michael G. Rodriguez, Joseph G. Pressey

**Affiliations:** Division of Hematology Oncology, University of Alabama at Birmingham, 2145 Bonner Way, Birmingham, AL 35243 USA; Department of Radiology, University of Alabama at Birmingham, 619 19th St South, Birmingham, AL 35249 USA; Department of Pediatrics, University of Alabama at Birmingham, 1600 7th Avenue South, Birmingham, AL 35233 USA; Cancer & Blood Disorders Institute, Cincinnati Children’s Hospital Medical Center, Cincinnati, OH USA

**Keywords:** Zoledronic acid, Bisphosphonates, Osteosarcoma, Metastatic, Survival, Human

## Abstract

**Background:**

Zoledronic acid (ZA) is a third-generation bisphosphonate in widespread clinical use to reduce pain and skeletal events in patients from a variety of malignancies with bone metastases. Pre-clinical studies indicate that ZA inhibits osteosarcoma through direct anti-proliferative effects, immune activation and anti-angiogenic activity.

**Methods:**

The purpose of this study was to evaluate the antitumor efficacy of ZA at standard dose until progression in patients with stage IV osteosarcoma lacking a standard of care treatment option proven to influence survival. Researchers retrospectively reviewed medical records of all patients at our institution with high-grade osteosarcoma presumed to be incurable due to metastases progressive after primary combination chemotherapy who received single agent ZA in an effort to delay progression.

**Results:**

In our four-patient cohort following initiation of ZA, the median progression-free survival was 19 months, and median overall survival was 56+ months. Two of four patients have remained progression-free since starting ZA. The other two initially progressed after 18–20 months on ZA followed by metastasectomy of lung or dural metastases and further stability for over a year following resumption of ZA. After a 20-month progression-free interval on ZA alone, one patient had partial response following addition of pazopanib to ZA that likely contributed to long term disease control. The four patients experienced no significant toxicities despite protracted dosing of ZA for up to 5 years, and none have required chemotherapy since beginning ZA.

**Conclusions:**

Single agent ZA was associated with encouraging progression-free survival in four consecutive patients with metastatic osteosarcoma. Prospective trials of single agent ZA are warranted as protracted maintenance therapy in surgically incurable osteosarcoma relapsed or refractory to first line combination chemotherapy with radiographically measurable metastases.

## Background

Osteosarcoma is the most common malignant bone tumor in children and young adults. Despite aggressive surgical and medical therapy, the estimated 5-year event-free survivals of patients presenting with localized or metastatic disease are 50–70 % and less than 20 %, respectively [1]. Patients with either unresectable primary tumors or distant metastases progressing after conventional primary chemotherapy have dismal outcomes, little changed over the past 30 years [2]. There is no “standard” second-line systemic therapy for inoperable osteosarcoma progressing after conventional chemotherapy. Results of three cooperative group experiences in this population reveal median survival of 6 months [3], median time to progression of 2 months with only 6 % event-free survival at 6 months [4], and a 2-year post-relapse survival <2 % [5]. Thus, novel systemic treatment approaches are urgently needed.

Zoledronic acid (ZA) is a third-generation, nitrogen-containing bisphosphonate analog of endogenous pyrophosphate that strongly binds to calcium-containing hydroxyapatite bone mineral. Although the plasma half-life of ZA is less than 24 h, bisphosphonates are stable in bone with a skeletal half-life of more than 300 days [6]. Osteosarcoma is characterized by the production of osteoid or immature bone containing focal calcium deposits of hydroxyapatite crystals [7]. Thus, ZA may preferentially concentrate in primary osteosarcomas as well as their lung and bone metastases and persist for years. ZA inhibits farnesyl diphosphate synthase, a key enzyme in the mevalonate pathway, and thus reduces protein prenylation essential for normal cell function and survival [8, 9]. Bone-resorbing osteoclasts internalize relatively large quantities of ZA and are thus particularly vulnerable to functional inhibition, underlying the widespread use of this drug to reduce pain and skeletal-related events in adults with bone metastases [10]. However, nitrogen-containing bisphosphonates appear to have direct anti-proliferative and pro-apoptotic effects against normal osteoblasts and a variety of tumor cells, including breast, myeloma, pancreatic and prostate cancers [11–15]. ZA has been shown to reduce primary tumor growth, decrease lung metastases and prolong survival in animal models of osteosarcoma [16–18]. ZA also acts synergistically with mTOR inhibition to decrease proliferation of murine and human osteosarcoma cell lines in vitro and to reduce osteosarcoma tumor growth in two distinct syngeneic murine modals [19]. Ewing’s sarcoma is the second most frequent malignant bone tumor in adolescents and young adults. ZA inhibits Ewing’s sarcoma cell invasion through down regulation of matrix metalloproteinase and significantly reduces development of spontaneous lung metastases in a nude mouse model of human Ewing’s sarcoma cells injected into the tibia [20].

The Children’s Oncology Group (COG) performed a phase I feasibility trial of monthly ZA for eight cycles with concurrent conventional multi-agent chemotherapy in 24 children with previously untreated, high-grade metastatic osteosarcoma [21]. The maximum tolerated dose (MTD) was equivalent to the standard adult dose for bone metastases, and there was a trend toward improved event-free survival at 2 years compared to historical data from intergroup study INT 0133 (*p* = 0.079). This manuscript is the first reported clinical experience with ZA as single agent therapy for osteosarcoma.

## Methods

With approval from The University of Alabama at Birmingham institutional review board, the researchers retrospectively reviewed medical records of all patients at our institution with high-grade osteosarcoma treated from December 2010 to January 2015. This timeframe was selected for analysis to allow a minimum of 8 months of follow-up for progression-free survival prior to this report. Twenty-seven such patients were identified; four of whom were presumed to be incurable due to metastatic osteosarcoma progressive after primary combination chemotherapy and received off-label ZA in an effort to delay progression. Those four consecutive patients so treated are the basis of this report. To provide details of each clinical situation and response to ZA, individual patient histories are briefly described in case report format. Treatment outcomes for the four patient cohort are summarized in Table 1.

**Table 1:** Treatment outcomes with zoledronic acid

Patient	Sites of disease^a^	PFS^b^ (months)	Additional therapy	Status at last visit	OS^b^ (months)
1	Lung	20	Pazopanib/surgery	NED	63+
2	Lung	54+	None	NED	54+
3	Lung and hilum	18	Radiation to hilum/neurosurgery	SD	59+
4	Lung	14+	None	SD	14+
		Median 19			Median 56+

*PFS* progression-free survival; *OS* overall survival; *NED* no evidence of disease; *SD* stable disease

^a^sites of osteosarcoma active during or immediately preceding ZA

^b^calculated from start of ZA

## Results

All four patients presented with high-grade osteosarcoma without detectable metastases. All received neoadjuvant and/or adjuvant chemotherapy with conventional doses of doxorubicin and cisplatin with or without high-dose methotrexate and ifosfamide. All underwent definitive surgical resection of the primary tumor using a limb-sparing approach in the three extremity cases. Patients 2 and 4 had microscopically positive soft tissue margins treated with post-operative external beam radiotherapy. All patients subsequently developed stage IV osteosarcoma involving the lungs that was treated with ZA. Patients 1 and 2 received ZA as consolidative treatment following metastasectomies and a likely transient response to salvage chemotherapy in the case of patient 2. Patients 3 and 4 received ZA for treatment of persistently measurable metastases to the lung and other sites. The University of Alabama at Birmingham Department of Anatomical Pathology histologically confirmed the diagnosis of high-grade osteosarcoma from the primary site of all four patients as well as from resection specimens from lung metastases in patient 1 before and after ZA and patient 2 before ZA. Histological confirmation of metastatic high-grade osteosarcoma was also obtained at our academic center from bronchoscopic biopsy of a hilar lymph node and subsequent resection of a brain metastasis in patient 3.

### Case #1

**Fig. 1:**
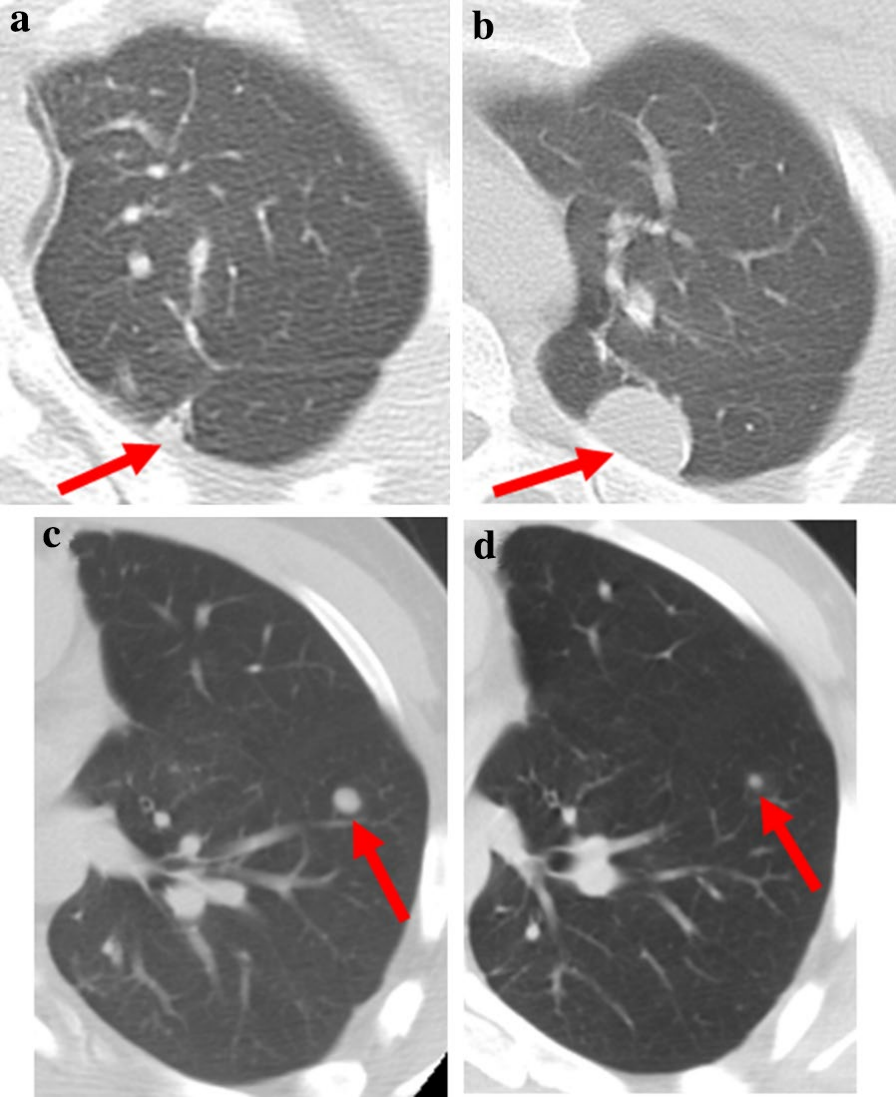
**a** Axial CT scan performed on 6/10/10 shows a new subpleural 6 mm *left* *upper lobe* nodule. **b** Follow-up CT performed on 12/10/10 shows interval increase in size of the pleural based *left upper lobe* nodule representing metastatic osteosarcoma at resection. **c** Axial CT performed on 3/28/11 shows a new 8 mm nodule in the *left lower lobe*. **d** Follow-up axial CT performed on 11/7/12 showed interval decrease in size of the *left lower lobe* nodule to 3 mm, confirmed to be osteosarcoma at subsequent resection

A 20-year-old male presented with osteosarcoma of the left tibia, and 16 months after completion of primary therapy a solitary metastasis of high-grade osteosarcoma was wedge resected from the right upper lobe. Over the subsequent 5 years prior to treatment with ZA, he required bilateral pulmonary metastasectomies on four occasions with no objective response to multiple intervening combination chemotherapy regimens including cyclophosphamide with topotecan, ifosfamide with etoposide, gemcitabine with docetaxel and cisplatin with vinorelbine. During that 5-year period, the longest progression-free interval was 6 months and the interval between metastasectomies progressively shortened from 20 months to 17 to 11 and ultimately to 9 months. A new subpleural 6 mm left upper lobe metastasis was seen on chest CT performed in June 2010 (Fig. 1a) and markedly enlarged on chest CT in December 2010 (Fig. 1b) prior to his fifth metastasectomy. In an attempt to delay further recurrence, ZA was initiated at 4 mg every 6 weeks rather than a 4-week interval indicated for bone metastases due to lengthy travel to clinic. Chest computed tomographies (CTs) were performed at 3-month intervals. After a 20-month progression-free interval, a solitary 8 mm metastasis to the left lung was detected (Fig. 1c). The patient continued ZA with the addition of oral pazopanib at 600 mg daily. The solitary metastasis responded to the combination of pazopanib and ZA, decreasing to 3 mm following 2 months of therapy. After 7 months, pazopanib was reduced to 400 mg daily due to plateaued response (Fig. 1d) as well as mild to moderate anorexia, insomnia and hypertension. After 14 months of combined pazopanib and ZA, chest computed tomography (CT) showed a stable 3 mm solitary left lung nodule, and ZA was discontinued to facilitate dental procedures related to advanced caries predating ZA use with no evidence of osteonecrosis. Pazopanib was also discontinued 4 months later to facilitate dental work with the lung nodule remaining stable after 18 months of pazopanib therapy. Chest CT after 3 months off therapy revealed enlargement of the left lung nodule from 3 to 17 mm, and positron emission tomography (PET) showed no disease elsewhere. The solitary left lung metastasis was wedge resected, and single agent ZA was resumed at the prior dose. The patient remains progression-free 20 months after surgery with overall survival 63+ months from the start of ZA.

### Case #2

A 63-year-old male underwent left radical parotidectomy and neck dissection to resect an extraosseous osteosarcoma with no lymph node involvement. Two years after completion of primary therapy a CT scan of the chest revealed a new right lung metastasis which was wedge resected. Repeat chest CT scan 3 months later demonstrated five new pulmonary metastases. He was considered incurable due to rapid progression of multiple, bilateral pulmonary metastases 2 years after completion of optimal adjuvant chemotherapy for a man of his age. He received salvage chemotherapy with four cycles of doxorubicin and dacarbazine since he was unable to tolerate additional cisplatin due to peripheral neuropathy. Chest CT scan showed a complete radiographic response which was followed by three cycles of vinorelbine with oral cyclophosphamide. Since the prognosis was dismal, the patient began ZA at 4 mg every 6 weeks due to lengthy travel to clinic as consolidative therapy. He continued this schedule for 18 months followed by dosing every 3 months for 36 additional months without apparent toxicity or relapse of osteosarcoma as determined by chest CT scans at 3-month intervals. He has been on ZA for 54+ months.

### Case #3

**Fig. 2:**
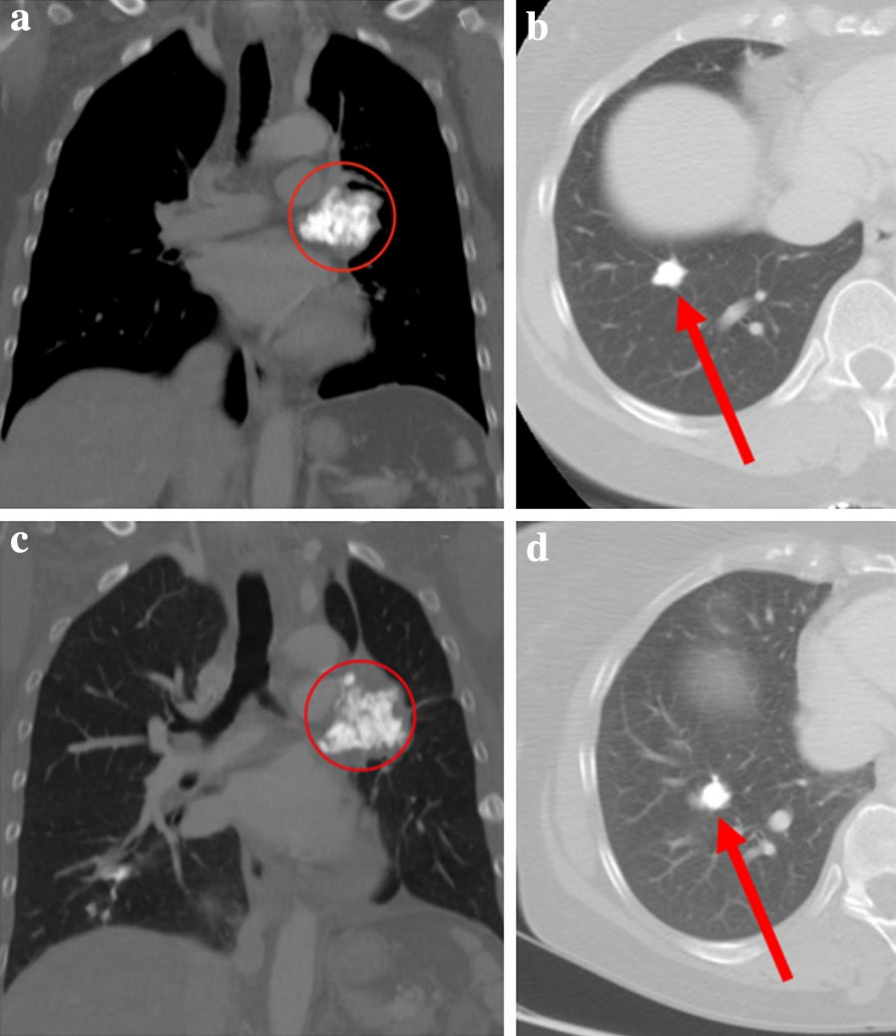
**a** Coronal CT image on 3/31/11 showing a calcified *left hilar* *mass* with metastatic osteosarcoma biopsy confirmed. **b** Axial CT image performed on 3/31/11 showing a calcified nodule in the *right lower lobe* consistent with metastatic disease. **c** Coronal CT image performed on 12/11/15 shows grossly stable appearance of the calcified *left hilar* *mass*. **d** Axial CT image performed on 12/11/15 shows a grossly stable appearance of the *right lower lobe* nodule

A 48-year-old female presented with osteosarcoma of the right femur, and 38 months after completion of primary therapy a chest CT scan revealed an enlarging 11 mm right lung nodule, new calcified left hilar adenopathy and narrowing of the lingular bronchus. Bronchoscopic biopsy confirmed metastatic osteosarcoma. Over the subsequent 19 months, she had no objective response to multiple chemotherapy regimens including ifosfamide with etoposide, cyclophosphamide with vinorelbine, gemcitabine, cisplatin and ridaforolimus. Bilateral pulmonary metastases increased in size and number, and left hilar adenopathy increased from 1.9 to 4.1 cm with distal atelectasis, cough and dyspnea on exertion (Fig. 2a, b). Since standard treatment options had been exhausted, ZA was initiated at 3.5 mg every 4 weeks due to an abnormal glomerular filtration rate at baseline. After 18 months of ZA, bilateral lung metastases remained stable, and the left hilar adenopathy increased modestly to 5 cm with progressive atelectasis. Although she was stable by RECIST 1.1 criteria, palliative radiation to 40 Gy in 8 fractions was delivered to the hilar mass to reduce dyspnea, and ZA was continued. After 26 additional months of ZA, the hilar adenopathy and multiple, bilateral pulmonary metastases remained stable by RECIST 1.1 criteria. A 5 cm intracranial dural-based calcified falcine mass was incidentally detected on sinus films, and neurosurgical resection revealed metastatic osteosarcoma. She has remained on monthly ZA for 59+ months and is stable 16 months after resection of the intracranial metastasis (Fig. 2c, d).

### Case #4

**Fig. 3:**
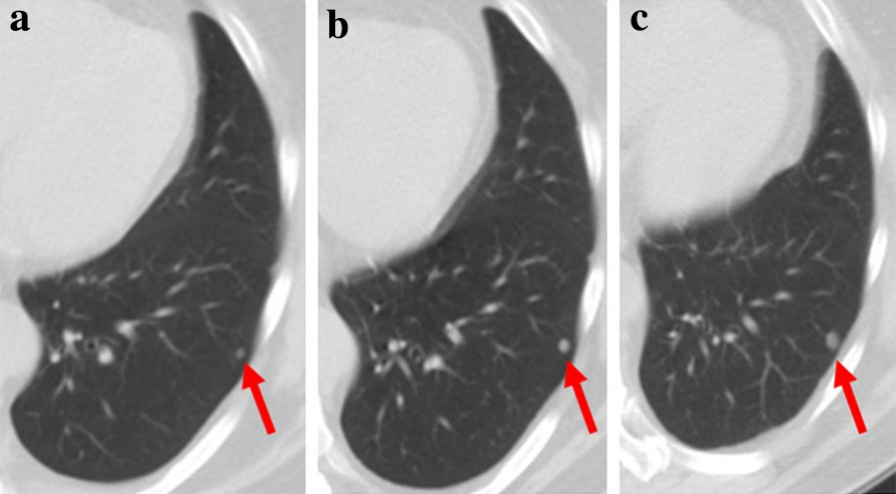
Axial CT images performed on 12/11/14 (**a**) and 1/23/15 (**b**) show interval growth of a *left lower lobe* nodule from 3 to 6 mm. Follow-up axial CT image performed on 2/1/16 (**c**) shows a grossly stable 6 mm *left lower lobe* nodule

A 65-year-old male presented with chondroblastic osteosarcoma of the left femur and multiple new bilateral lung metastases measuring up to 1 cm diameter developed during the fourth cycle of adjuvant chemotherapy. These were confirmed to be progressive by two CT scans of the chest performed 6 weeks apart (Fig. 3a, b). Chemotherapy was discontinued, and ZA was initiated at 4 mg every 4 weeks. After 14 months of follow-up, the patient remains stable with no local recurrence, pulmonary metastases stable by RECIST 1.1 criteria (Fig. 3c) and no new lesions as determined by MRI and chest CT, respectively at 3 month intervals.

### Summary of outcomes

All patients had stage IV osteosarcoma and lacked a standard of care treatment option proven to significantly influence survival. The treatment outcomes with ZA are summarized in Table 1. Following initiation of ZA, the median progression free survival was 19 months, and median overall survival was 56+ months. At the time of this report, two of the four patients have no evidence of disease, and the other two have stable disease. None of the four patients have required chemotherapy since starting ZA. The case of patient 1 is particularly compelling since ZA dramatically extended the progression-free interval following his fifth pulmonary metastasectomy to 20 months. Once another metastasis became detectable, it objectively responded to the addition of pazopanib to ZA for 14 months followed by resection, and he remains progression-free 20 months later on single agent ZA. Patient 3 demonstrated stable hilar and pulmonary metastases for a remarkable 44 months on ZA allowing her to survive long enough to develop a very uncommon metastasis to the dura possibly representing a sanctuary site of relatively poor drug penetration. Following resection of the intracranial metastasis, she has remained stable for an additional 16 months on ZA.

## Discussion

Bisphosphonates are relatively well tolerated with side effects including electrolyte disturbances, flu-like symptoms, nausea and less commonly osteonecrosis of the jaw and renal dysfunction [22, 23]. The four patients reported herein receiving ZA for advanced osteosarcoma experienced no significant toxicities despite protracted dosing for up to 5 years. ZA is a convenient treatment for patients with incurable osteosarcoma with far less detrimental impact on quality of life than conventional chemotherapy.

Pre-clinical studies indicate that ZA inhibits osteosarcoma through direct anti-proliferative effects, immune activation and anti-angiogenic activity. ZA directly inhibits human osteosarcoma cell proliferation and induces apoptosis in vitro [24–26]. γδ T cells recognize small phosphoantigens associated with metabolites of bacterial isoprenoid biosynthesis or the mevalonate pathway in eukaryotes [27]. ZA sensitizes tumor cells to γδ T cell cytotoxicity through accumulation of mevalonate pathway intermediates within bisphosphonate-treated cells [28]. ZA promotes γδ T cell proliferation from peripheral blood mononuclear cells of osteosarcoma patients and enhances γδ T cell cytotoxicity against human osteosarcoma cells [29, 30]. Vascular endothelial growth factor-A (VEGF-A) and vascular endothelial growth factor receptor-1 (VEGFR-1) are highly expressed by aggressive osteosarcoma cells, but not in clonally-related less aggressive cell lines [31]. Plasma levels of VEGF-A are elevated in osteosarcoma patients, and ZA decreases circulating levels of VEGF-A in these patients [32, 33]. ZA inhibits tumor-associated angiogenesis not only via direct inhibition of endothelial cells, but also by decreasing VEGF-A expression in osteosarcoma. Furthermore, evidence indicates that ZA induces apoptosis in aggressive osteosarcoma cells at least partly by inhibiting autocrine stimulation via VEGF-A and VEGFR signaling [31]. Thus, nitrogen-containing bisphosphonates have pleiotropic inhibitory effects on osteosarcoma and tumor-associated endothelial cells.

Inherent limitations in this study include the retrospective nature of the review and the small cohort of patients analyzed. However, this report does include all patients receiving single agent ZA to delay osteosarcoma progression at our institution during the specified timeframe.

COG has demonstrated the feasibility of co-administering ZA with conventional chemotherapy for first line treatment of metastatic osteosarcoma, but the small trial was underpowered to detect efficacy of ZA, particularly in the presence of highly active chemotherapy. A large, randomized French trial of conventional adjuvant chemotherapy with or without ZA for resectable primary osteosarcoma was stopped early due to futility with hazard ratios for event-free survival and death exceeding unity in the ZA cohort. Although not statistically significant, these data suggests that ZA in this context may increase mortality [34]. However, this trial may likewise have been suboptimally designed to evaluate efficacy of ZA in this disease. Direct antiproliferative effects of ZA could actually protect osteosarcoma cells from tumoricidal effects of chemotherapy. Enhancement of antitumor immune responses by ZA could be undermined by concurrent cytotoxic chemotherapy, and antiangiogenic actions of ZA may have little relevance in treating micrometastases in the adjuvant setting. ZA may actually have greater efficacy against surgically incurable macroscopic osteosarcoma used as a single agent or in combination with the mTOR inhibitor everolimus or the VEGFR tyrosine kinase inhibitor pazopanib, targeting tumor cell proliferation and angiogenesis, respectively [19, 35]. The combination of ZA and sirolimus has recently been demonstrated to be effective and tolerable therapy for a young adult with aggressive lymphatic malformations associated with Gorham-Stout disease [36]. Intriguing results from a recent study of the multi-tyrosine kinase inhibitor sorafenib with activity against VEGFR in combination with everolimus further support the use of such agents in recurrent or refractory osteosarcoma [37]. In fact, this trial represents the first positive study in at least 25 years in patients with relapsed osteosarcoma [38]. Interestingly, patient 1 in this report experienced disease control for 20 months on single agent ZA followed by emergence of another lung metastasis that objectively responded for over 14 months to the addition of pazopanib to ZA. We have also observed objective response of multiple pulmonary metastases to combined ZA and pazopanib for over 9 months in a patient with relapsed osteosarcoma not included here due to lack of single agent ZA use.

## Conclusions

Zoledronic acid represents a convenient, well tolerated therapy which may provide durable control of incurable osteosarcoma. Therefore, prospective trials of single agent ZA are warranted in surgically incurable osteosarcoma relapsed or refractory to first line conventional combination chemotherapy with radiographically measurable metastases.

## Abbreviations

ZA: zoledronic acid
mTOR: mammalian target of rapamycin
CT: computed tomography
PET: positron emission tomography
RECIST: response evaluation criteria in solid tumors
MRI: magnetic resonance imaging
VEGF: vascular endothelial growth factor
VEGFR: vascular endothelial growth factor receptor
COG: Children’s Oncology Group
